# Artery of Percheron Strokes: Three Cases in Three Months

**DOI:** 10.7759/cureus.21688

**Published:** 2022-01-28

**Authors:** Julianne Flowers, Sani Gandhi, Lakshmi Guduguntla, Alexander Yang, Shyam Moudgil

**Affiliations:** 1 Neurology, Wayne State University School of Medicine, Detroit, USA; 2 Neurology, Rutgers Robert Wood Johnson Medical School, New Brunswick, USA; 3 Neurology, Ascension Saint John Hospital, Detroit, USA

**Keywords:** tpa, cva, percheron, stroke, thalamus

## Abstract

The artery of Percheron (AOP) is a rare variant of thalamic vasculature and is a single dominant thalamoperforating artery supplying bilateral paramedian thalamic territories. Occlusion of the AOP results in a characteristic pattern of bilateral paramedian thalamic infarcts and is estimated to represent between 0.1%-0.3% of all ischemic strokes and 4% to 35% of all thalamic strokes. Four distinct ischemic patterns of AOP infarcts have been identified: bilateral paramedian thalamic region with midbrain (43%), bilateral paramedian thalamic without midbrain (38%), bilateral paramedian thalamic with anterior thalamus and midbrain involvement (14%), and bilateral paramedian thalamic with anterior thalamus without midbrain involvement (5%).

Despite our knowledge of the characteristic radiologic features of an AOP stroke, the true incidence of AOP strokes is challenging to estimate due to non-specific clinical symptoms and subtle findings on computed tomography (CT) and/or magnetic resonance imaging (MRI). Here, we present a case series of three patients seen within a 3-month span at one community hospital seen by one single neurologist with confirmed AOP stroke by radiologic imaging. The frequency of these cases suggests that the incidence of AOP infarctions may be higher than previously estimated and instead are underreported due to broad differential on clinical and imaging presentation.

## Introduction

With an estimated incidence of 4-12% of the population, the artery of Percheron (AOP) is a rare variant of neurovascular supply to the thalami and midbrain [1]. The AOP arises from the posterior cerebral artery (PCA) and supplies blood to the paramedian and the rostral midbrain [2]. Four distinct ischemic patterns of AOP infarcts have been identified: bilateral paramedian thalamic region with midbrain (43%), bilateral paramedian thalamic without midbrain (38%), bilateral paramedian thalamic with anterior thalamus and midbrain involvement (14%), and bilateral paramedian thalamic with anterior thalamus without midbrain involvement (5%) [1]. Clinical symptoms of an AOP infarct include altered mental status (AMS), memory impairment, and vertical gaze palsy. Radiologic characteristics on magnetic resonance imaging (MRI) include the “V-sign”, a hyperintense signal along the midbrain adjacent to the interpeduncular fossa, which is 67% sensitive for an AOP infarction with midbrain involvement [1]. These non-specific symptoms and radiologic signs combined with the rarity of the AOP variant make diagnosing AOP infarcts a particularly challenging task for clinicians. Here, we describe three cases of confirmed AOP infarcts seen within a 3-month period.

## Case presentation

Case 1

The patient was a 63-year-old woman with a past medical history of hypertension, chronic obstructive pulmonary disease, obesity, diabetes mellitus, and hyperlipidemia who presented with AMS. Three days prior to admission, she was observed by her granddaughter as being lethargic and drowsy. Labs were significant for leukocytosis, and urinalysis tested positive for white blood cells, nitrates, leukocyte esterase, and blood. Thus, this initial work-up suggested AMS secondary to urinary tract infection, and the patient was treated empirically with ceftriaxone and vancomycin.

**Figure 1:**
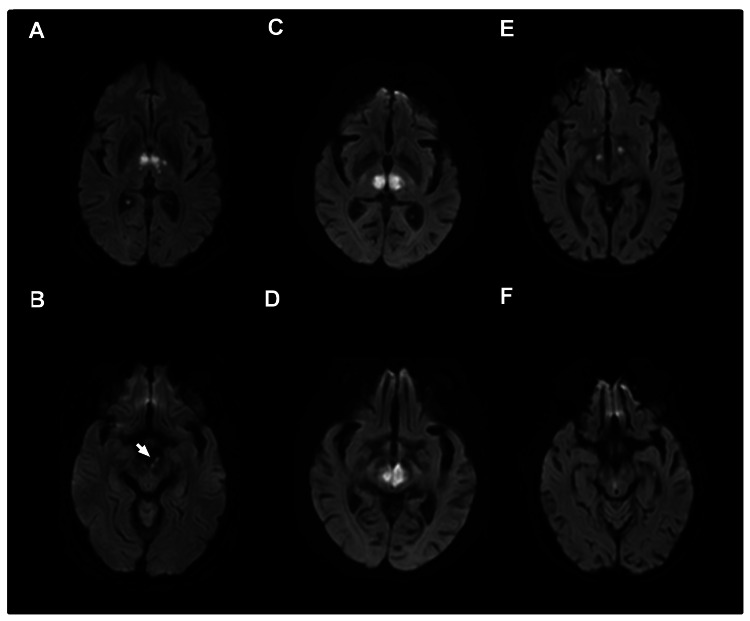
Representative radiologic appearance of artery of Percheron infarctions using magnetic resonance diffusion-weighted imaging in each of the three patients. A, Bilateral medial thalamic infarcts with B, positive “V sign”-hyperintensity along the midbrain adjacent to the interpeduncular fossa. C, Acute ischemic infarcts found in bilateral medial thalami and acute infarcts found in the D, left midbrain and right paramidline midbrain. E, Acute infarcts bilateral thalami and F, right paramedian of the midbrain.

However, the next day neurology was consulted due to persistent AMS and recommended MRI, which revealed acute infarction of the medial thalamus and a positive “V sign”, hyperintensity along the midbrain adjacent to the interpeduncular fossa (Figures 1a-1b). On day three of admission, vascular consultation and neurological consultation noted marked improvement of alertness and recommended outpatient follow-up and subacute rehabilitation. The patient was discharged on the sixth day of admission after significant improvement in mentation.

Case 2

The patient was a 90-year-old Caucasian female with a past medical history of hypertension, advanced Alzheimer’s disease, type 2 diabetes, recurrent urinary tract infections with extended-spectrum beta-lactamase (ESBL), and major depressive disorder, who presented with AMS from an extended nursing facility. On presentation, the patient had a Glasgow Coma Scale score of 8, and vital signs were notable for hypothermia with a temperature of 91.6 °F, which increased to 93.4 °F with external rewarming. Electrocardiogram revealed sinus bradycardia with no signs of arrhythmia. CT Head without contrast was obtained and revealed the absence of acute intracranial hemorrhage or infarcts, but did show several leukodystrophic plaques compatible with chronic microvascular changes. Urinalysis and urine culture was obtained and were positive for pan-sensitive *E. coli* and the patient was started on ceftriaxone. Her working diagnosis was metabolic encephalopathy secondary to urosepsis.

However, after three days of therapy, the patient’s mental status did not improve, so the neurology team was then consulted. MRI revealed large acute infarcts in the medial aspect of bilateral thalami along with acute infarcts found in the left midbrain and right paramidline midbrain (Figures 1c-1d). After five days of hospitalization, the family made a decision to transfer the patient to hospice.

Case 3

The patient was a 77-year-old man with a past medical history of cerebral toxoplasmosis and HIV who presented after a fall at home and AMS. The patient also complained of right-sided chest wall and abdominal pain that was reproducible upon palpation. CT Head and angiogram revealed a left middle cerebral artery aneurysm but with no evidence of acute hemorrhage. Urinalysis was positive for leukocyte esterase but was negative for nitrates and blood. Rapid urine drug screen was positive for opiates, and naloxone was administered with minimally positive effect. Encephalopathy in the setting of positive opioid use and central nervous system infection were the two top differential diagnoses given the patient’s previous medical history.

On the second night of admission, the patient's blood pressure increased to 207/89 and was controlled with clonidine and lisinopril. On the third day of admission, EEG demonstrated mild diffuse encephalopathy with no clear abnormalities. MRI was performed and revealed bilateral thalamic infarcts and right paramedian midbrain (Figures 1e-1f). As a result, the patient was continued on his home clopidogrel and started on atorvastatin. Speech language determined a moderate cognitive-linguistic impairment with moderate dysarthria with conversational intelligibility at 50% and fluctuating alertness throughout the exam. The patient was discharged to subacute rehabilitation after repeat CT was negative for any additional acute neurologic processes.

## Discussion

The incidence of AOP strokes has been estimated to be 0.1-0.3% of all ischemic strokes and as high as 2% of all ischemic strokes [3,4]. A previous retrospective chart review identified 12 AOP infarct patients in a 17-year period at one institution [5]. We identified three patients at a local community hospital in a 3-month span, suggesting the incidence of AOP variant is more common in our region. Two out of three of our patients were African American (AA), however, there are currently no studies to suggest variations in the predominance of the AOP variant within different ethnic groups. Lastly, all three patients had the most common anatomic pattern of AOP - bilateral thalamic paramedian with midbrain involvement [1].

Classic symptoms include mental status changes and vertical gaze paresis, which overlap with other neurological disorders such as Wernicke-Korsakoff syndrome and toxic-metabolic encephalopathy [5]. Two out of three patients had hyperlipidemia and hypertension, risk factors for stroke, however, the etiology of the strokes for our patients was unknown. Importantly, all three patients were seen before the COVID-19 pandemic and thus were not affected by COVID-19. Although all patients presented with altered mental status, confusion was attributed most often to underlying infection such as urinary tract infection (UTI), upper respiratory infection (URI) with sepsis, or central neurological system (CNS) re-infection as in Case 3 due to history of cerebral toxoplasmosis in the setting of HIV (Table 1). Due to the lack of classic stroke presentations in addition to more identifiable causes of mental status changes, alternative diagnoses remained higher on the differential, and as a result, only one patient was suspected of having a stroke on initial workup. Radiologically, AOP strokes are difficult to diagnose with routine stroke imaging such as CT head and/or CT angiogram of the head [1]. In our cohort, CT scans for all three patients were negative for any hemorrhagic etiology and required the use of MRI to finalize the diagnosis of AOP stroke. On average, MRI was performed 60 hours after admission to confirm cerebral infarct with bilateral thalamic involvement. In our cohort, none of the patients were diagnosed timely enough for tissue Plasminogen Activator (tPA) intervention. One of the patients was dispositioned to hospice, while the other two were dispositioned to subacute rehabilitation (Table 1).

**Table 1:** Presenting Symptoms and Characteristics of Patients Abbreviations: F, female, M, male, AA, African American, HTN, hypertension, DM, diabetes mellitus, HLD, hyperlipidemia, HF, heart failure, UTI, urinary tract infection URI, upper respiratory infection, BG, basal ganglia, SAR, subacute rehabilitation, CNS, central neurological system, h, hours, d, days, Dx, Diagnosis, DDX, differential diagnosis, CVA, cerebral vascular accident, FHx, family history, CAD, coronary artery disease

Patient	Sex/Race/Age	Risk Factors	Optical Symptoms	Language Dysfunctions	Time to Dx	Brain Findings (in addition to thalamic infarct)	Disposition	DDx
1	F, AA,63	HTN, obesity, DM, HLD, falls, FHx mom CVA, obesity	None	Dysphagia-regular solids, thin liquids, dysarthria, moderate mixed receptive and expressive aphasia, dyslexia, dysgraphia	26 hrs	Midbrain adjacent to interpeduncular fossa	SAR	Encephalopathy likely secondary too UTI or flexeril use
2	F, White,90	HTN, Alzheimer's, DM2, HF, past UTI, CAD	Pupils 1mm and minimally reactive	Severe oral dysphagia, no response	3d	Acute infarct left midbrain with small right paramidline midbrain involvement. Tiny left cerebellar acute infarct.	Nursing home hospice	Encephalopathy likely due to urosepsis
3	M, AA,77	Cerebral toxoplasmosis previous year, HIV	None	Slow monotonous speech, dysphagia III/MS chopped diet thin liquid, moderate dysarthria,	3d 10hrs	Small right paramedian infarct of the midbrain	SAR	Encephalopathy unknown etiology opioids or CNS infection

In the previous cohort of 12 patients in the previous University of Michigan study, all but one had residual deficits on discharge [5]. The one patient without residual neurologic deficits on discharge received prompt intervention with tPA [5]. Other therapeutic strategies such as endovascular revascularization are difficult due to the small diameter of the AOP [6]. Because of our small retrospective sample size, we cannot precisely identify the factors that resulted in delays in diagnosis and intervention with tPA. Thus, earlier suspicion for AOP infarct in our patients may have resulted in prompt intervention and better outcomes. Our case series reminds physicians the incidence of AOP strokes may be higher than previously estimated and to include this rare variant on the differential for mental status changes that are refractory to treatment.

## Conclusions

Because of our small retrospective sample size, we cannot precisely identify the factors that resulted in delays in diagnosis and intervention with tPA. Thus, earlier suspicion for AOP infarct in our patients may have resulted in prompt intervention and better outcomes. Our case series reminds physicians the incidence of AOP strokes may be higher than previously estimated and to include this rare variant on the differential for mental status changes that are refractory to treatment.
